# NerveTracker: a Python-based software toolkit for visualizing and tracking groups of nerve fibers in serial block-face microscopy with ultraviolet surface excitation images

**DOI:** 10.1117/1.JBO.29.7.076501

**Published:** 2024-06-18

**Authors:** Chaitanya Kolluru, Naomi Joseph, James Seckler, Farzad Fereidouni, Richard Levenson, Andrew Shoffstall, Michael Jenkins, David Wilson

**Affiliations:** a Case Western Reserve University, Department of Biomedical Engineering, Cleveland, Ohio, United States; b UC Davis Medical Center, Department of Pathology and Laboratory Medicine, Sacramento, California, United States; c Louis Stokes Cleveland VA Medical Center, Cleveland, Ohio, United States; d Case Western Reserve University, Department of Pediatrics, Cleveland, Ohio, United States; e Case Western Reserve University, Department of Radiology, Cleveland, Ohio, United States

**Keywords:** tractography, peripheral nerves, optic flow, structure tensor analysis, microscopy

## Abstract

**Significance:**

Information about the spatial organization of fibers within a nerve is crucial to our understanding of nerve anatomy and its response to neuromodulation therapies. A serial block-face microscopy method [three-dimensional microscopy with ultraviolet surface excitation (3D-MUSE)] has been developed to image nerves over extended depths *ex vivo*. To routinely visualize and track nerve fibers in these datasets, a dedicated and customizable software tool is required.

**Aim:**

Our objective was to develop custom software that includes image processing and visualization methods to perform microscopic tractography along the length of a peripheral nerve sample.

**Approach:**

We modified common computer vision algorithms (optic flow and structure tensor) to track groups of peripheral nerve fibers along the length of the nerve. Interactive streamline visualization and manual editing tools are provided. Optionally, deep learning segmentation of fascicles (fiber bundles) can be applied to constrain the tracts from inadvertently crossing into the epineurium. As an example, we performed tractography on vagus and tibial nerve datasets and assessed accuracy by comparing the resulting nerve tracts with segmentations of fascicles as they split and merge with each other in the nerve sample stack.

**Results:**

We found that a normalized Dice overlap (${\text{Dice}}_{\text{norm}}$) metric had a mean value above 0.75 across several millimeters along the nerve. We also found that the tractograms were robust to changes in certain image properties (e.g., downsampling in-plane and out-of-plane), which resulted in only a 2% to 9% change to the mean ${\text{Dice}}_{\text{norm}}$ values. In a vagus nerve sample, tractography allowed us to readily identify that subsets of fibers from four distinct fascicles merge into a single fascicle as we move ∼5  mm along the nerve’s length.

**Conclusions:**

Overall, we demonstrated the feasibility of performing automated microscopic tractography on 3D-MUSE datasets of peripheral nerves. The software should be applicable to other imaging approaches. The code is available at https://github.com/ckolluru/NerveTracker.

## 1 Introduction

Peripheral neuromodulation is a growing area of research that involves the application of external stimuli to modulate nerve activity.^1^ Although these methods have been applied to treat various disease conditions, the effectiveness of these therapies has been limited. For instance, vagus nerve stimulation (VNS) is a clinically approved therapy to treat conditions such as drug-resistant epilepsy,^2^ depression,^3^ and obesity.^4^ However, therapeutic efficacy with this method is just moderate, with 50% of patients seeing a significant (>50%) reduction in seizure frequency after 1 year of implantation.^5^ In addition, several trials involving VNS therapy have failed to reach clinical endpoints.^6^^,^^7^ We find that computational modeling approaches can aid researchers to test various interface designs and identify ideal stimulation parameters (pulse amplitude, width, duty cycle, etc.); these could serve to improve fiber selectivity and thereby increase therapeutic efficacy. However, these models require accurate anatomical information about human nerve morphology since the latter significantly affects optimal stimulation parameters.^8^ Therefore, there is a need for methods that enable visualization and mapping of human peripheral nerves to improve computational models, providing a deeper understanding of nerve responses to neuromodulation therapies and enhancing treatment outcomes.

While prior studies have described the macroscopic connectivity of peripheral nerves in detail, reports on the arrangement of fibers within fascicles along the length of the nerve have been limited thus far.^9^^–^^11^ The analysis is also challenging in nerves such as the vagus, which can be over 40 cm in length in adults.^10^ We are developing image acquisition and analysis methods to track groups of fibers along the length of a peripheral nerve to determine how they split, merge, and coincide with other fiber tracts.

To image peripheral nerves at fiber resolution, several methods have been proposed. The gold standard methods to image nerve anatomy involve electron microscopy (EM), including transmission EM for two-dimensional (2D) imaging of thin sections and serial block-face EM for three-dimensional (3D) imaging.^12^^,^^13^ Advances in multiphoton and super-resolution microscopy methods have also been described to image peripheral nerves with and without labels.^14^ However, these systems are appropriate for very small volumes and are technically complex and expensive. Diffusion MRI approaches have been developed to image white matter tracts in the human brain *in vivo*.^15^^–^^17^ These methods are also being used clinically to evaluate various peripheral nerve injuries, including traumatic lesions^18^ and chronic neuropathies.^19^ Recent preclinical advances have made these methods suitable for microscopic imaging of *ex-vivo* tissues as well, including mouse brain^20^ and postmortem human spinal cord.^21^ However, resolution and contrast in routine diffusion MRI datasets limit direct visualization of fiber structure within peripheral nerves, thereby requiring additional validation methods. Micro-CT using a conventional x-ray source with appropriate tissue staining has been used to image nerve structure *ex vivo*.^11^^,^^22^ These methods have shown that some nerves (e.g., vagus^18^) have more fascicle branching than previously appreciated. However, this technique is limited to the visualization of nerve fascicles rather than nerve fibers. To address these technical challenges, we have developed a serial block-face imaging system based on the microscopy with ultraviolet surface excitation (MUSE) technique.^23^^,^^24^ MUSE is based on the use of fluorescent stains, possibly complemented by absorbing stains for additional contrast, excited using short-wavelength (280-nm range) ultraviolet light that penetrates only ∼10 to 20  μm below the surface of tissue specimens.^25^ This simplifies the setup by obviating the need for alternative and more complex optical sectioning methods, as in confocal microscopy. The application of MUSE in a serial block-face imaging setup permits imaging of samples across extended depths and has been described in previous reports.^24^^,^^26^^–^^29^

In our approach, referred to as 3D-MUSE, nerve samples are first stained with an absorptive dye to delineate structures of interest and then counterstained with an ultraviolet fluorescent dye (commonly rhodamine), followed by dehydration and embedding into resin blocks for section and imaging. We adopted a previously published method for whole-mount staining of nerves in cadaver tissues called Sihler’s staining.^30^ 3D-MUSE is suitable for imaging embalmed human cadaver nerves.

3D-MUSE datasets of peripheral nerve tissues can be analyzed with tractography methods to build a map of the underlying fiber organization, which could be a step toward building a connectome for the peripheral nervous system. Although imaging methods based on light microscopy do not commonly have sufficient resolution to clearly resolve all nerve fibers (e.g., unmyelinated fibers can be under 0.5  μm in diameter), tractography methods can still be used to estimate fiber orientation. The methods assume that the underlying displacement field is smooth and estimate flow vectors by analyzing a small neighborhood around each pixel. The flow vectors serve as a proxy for fiber displacements and can be used to build a tractogram starting from a defined set of seed points. Similar methods to estimate the orientation of fiber groups have been applied in a wide range of datasets, including diffusion MRI,^17^ microscopy,^31^ and OCT.^32^ In the context of processing diffusion MRI data, several tractography toolkits are publicly available, including Diffusion Toolkit,^33^ Slicer DMRI,^34^ DSI Studio,^35^ and DIPY.^36^ However, these software tools are made to work with modality-specific datasets (e.g., diffusion-weighted images acquired at various gradient strengths). Modifying them to work with microscopy data is not straightforward, suggesting the need to create a dedicated software tool. In addition, there is an opportunity to create a specialized solution for peripheral nerves with their long-axis geometry and fascicles surrounded by fiber-free epineurium.

In this work, we developed a software package, NerveTracker, to visualize and track groups of nerve fibers in 3D-MUSE datasets. The software is implemented as a multi-threaded application in Python and is built on top of the Qt and VTK libraries. The software utilizes either optic flow or structure tensor algorithms to determine local fiber orientation. Tracking is initialized by color-seeding each group of fibers within a specific slice of a 3D stack. Tracking follows the orientation field in either direction along the stack. The resultant set of streamlines (referred to as a tractogram) can be interactively visualized and edited. Using deep learning, we segmented fascicle regions and anatomically constrained streamlines to stay within the mask. Novel metrics to measure the correspondence of a tractogram with ground truth fascicle (groups of fibers) segmentation as well as similarity values for comparing two tractograms are used. Example results are shown.

## 2 Image Analysis Methods

### 2.1 Preprocessing

Prior to running a tractography algorithm on the image stack, a mask image for placing seed points is manually created in the software. The mask should label regions of interest (ROIs) on a specific slice in the stack. Alternatively, the mask can be generated by an automated segmentation algorithm as described later in Sec. 2.4. The software generates seed point coordinates by randomly sampling within the foreground regions of the mask. In addition, a few preprocessing steps are performed on the image stack. In a typical 3D-MUSE imaging setup, images are captured using a color camera, hence containing RGB color information. However, to reduce memory requirements and processing times, images are converted to grayscale by computing a weighted sum of the individual color channels (50:50 red and green). An optional gamma correction step can be applied to further improve image contrast.

### 2.2 Flow Estimation

The software includes two flow estimation algorithms suitable for block-face microscopy datasets. One approach utilizes the optic flow algorithm,^37^ a popular object tracking algorithm in computer vision. The second approach involves structure tensor analysis,^38^ a method for determining fiber orientation in microscopy data.

The optic flow method can be applied to our 3D MUSE datasets. Since our images are transverse to the nerve, a large proportion of fibers are oriented approximately perpendicular to the imaging plane. This allows us to consider the 3D image stack as a sequence of video frames and formulate the problem as tracking points from one image frame to the next. We utilize the Lucas–Kanade algorithm^39^ to estimate flow vectors at all pixels of interest. The method solves the optic flow in Eq. (1) at a given pixel by assuming that the flow is constant within a neighborhood (say n×n  pixels, where n is a user-defined parameter). This results in a system of $n^{2}$ linear equations and two unknown flow variables, resulting in an overdetermined system. A least-squares approach is used to find the best fit. In Eq. (1), $I_{x}$ (p), $I_{y}$ (p), and $I_{z}$ (p) indicate derivatives calculated along the X, Y, and Z axes, respectively. In our case, the Z axis of the image stack can be considered equivalent to the time axis in traditional optic flow methods for object tracking in videos. $V_{x}$ and $V_{y}$ indicate the magnitude of the flow vectors in the X and Y axes, respectively

$$I_{x}(p)V_{x}+\;I_{y}(p)V_{y}=\;-I_{z}(p).$$ (1)

The above equation is derived under two assumptions. First, we assume that the brightness of the tracked objects does not change from one frame to the next. This is a reasonable assumption since we consider two consecutive images in the stack. Second, the displacements between images are assumed to be small, allowing one to use a linear approximation in the model. Also, we utilize a pyramidal model^40^ to account for cases where the displacement field is not small. In this case, images are downsampled multiple times, and an initial flow estimate is computed on data with the lowest sampling rate (largest pixel size). The resultant flow fields can be assumed to be small and are used to transform images at the next largest sampling rate prior to computing flow again. The process is repeated until the original image sampling rate is reached. The number of downsampling steps is passed as a user-defined parameter to the algorithm, and the downsampling occurs by a factor of two at each level.

In addition to the optic flow approach, an orientation estimation method based on structure tensor analysis^31^^,^^38^ is implemented in the proposed software to estimate flow. The method computes a 3D orientation vector at each voxel in the stack. Formally, we find the vector u at each voxel that minimizes the quantity shown in Eq. (2)

$$D=\underset{p^{\prime }\in N(p)}{\sum }{\left(V(p^{\prime }+u)-V(p^{\prime })\right)}^{2}.$$ (2)

In the equation shown above, p represents the current voxel of interest at location (x,y,z) and N(p) indicates a neighborhood of voxels centered at p (size specified as a user-defined parameter). $V(p^{\prime })$ indicates the image intensity value at voxel $p^{\prime }$ located in the neighborhood N(p). The metric D can be interpreted as follows. For a given vector u, the algorithm displaces the neighborhood N(p) in the direction of u. The sum of squared differences in pixel intensities in the neighborhood before and after the displacement equals the metric D, which is minimized. Using Taylor series approximations, the metric is described in terms of a 3×3 structure tensor composed of partial derivatives in x, y, and z ($V_{x}$, $V_{y}$, and $V_{z}$, respectively) and the direction vector u. This form of the metric is shown in Eq. (3). Subsequently, Eigen decomposition is performed on the tensor, and the eigenvector with the smallest eigenvalue is identified as the vector that results in a minima for the metric D. This vector is the orientation vector, which is fed as input to generate streamlines

$$D=u^{T}\underset{p^{\prime }\in N(p)}{\sum }[\begin{array}{c} V_{x}(p^{\prime })\\ V_{y}(p^{\prime })\\ V_{z}(p^{\prime }) \end{array}][\begin{array}{ccc} V_{x}(p^{\prime }) & V_{y}(p^{\prime }) & V_{x}(p^{\prime }) \end{array}]u.$$ (3)

The methods described above provide an estimate of flow at image pixels. The optic flow algorithm assumes that the motion occurs from one frame to the next and provides x and y displacements at a given pixel. These values can be added to a pixel’s coordinates to find its new location in the subsequent frame. Streamlines are drawn by connecting the old and new pixel coordinates and repeating this process in an iterative fashion in either direction along the stack. In the structure tensor approach, each pixel receives a 3D unit vector corresponding to local fiber orientation. We scale this vector to find its intersection with the next image plane and record its coordinates as the new location of the pixel in the next frame. Subsequently, streamlines can be drawn connecting the old and new pixel locations, as done in the optic flow approach. In both approaches, the new locations for the tracked pixels can land in between image pixel coordinates with sub-pixel accuracy; thus, an interpolation step is necessary to determine the subsequent flow field. For the structure tensor analysis, nearest neighbor interpolation is used. In addition, tracking is stopped in both approaches if the streamline diverges by an angle greater than 75 deg with respect to the long axis of the nerve, similar to termination conditions in diffusion MR tractography. Individual streamlines are colored based on the location of their seed points in the binary mask input by the user, which may contain one or more connected components. In general, each fascicle can be delineated as a unique connected component (ROI). All streamlines originating from a specific ROI in the mask are given a unique color for visualization.

### 2.3 Tractogram-Based Metrics

NerveTracker implements two metrics to evaluate tractograms generated by the methods described above. The first metric measures the accuracy of a tractogram by assessing its overlap with ground truth segmentations. To accomplish this, the tractogram can be considered a set of 2D point clouds, with one 2D point cloud on each slice of the stack. This makes it straightforward to compare with corresponding segmentations at the same slice. We convert the tractogram point cloud on a slice into a binary mask by finding the nearest pixel coordinate for each point in the point cloud and setting it to be a part of the foreground mask. Since streamlines originating from a particular ROI in the user-provided mask can be identified based on their unique color, individual tractogram masks can be created for each ROI in the mask. Next, to assess the accuracy of streamlines, ground truth segmentations are created by identifying fiber groups within each ROI, tracking them visually through the image stack, and outlining them on intermediate slices. This process generates a ground truth mask for comparison. Finally, the Dice metric can be used to calculate the overlap between the binary mask derived from the tractogram and the ground truth mask for each ROI. Since an increase in the seed point density on the starting slice may result in a higher Dice coefficient due to a corresponding increase in the number of points in the point cloud, we normalize the computed Dice coefficients with respect to the Dice coefficient on the starting slice. We refer to this metric as ${\text{Dice}}_{\text{norm}}$. Although the Dice coefficient is an area-based metric, it is reasonable to compare its value across slices under the assumption that the overall cross-sectional area of fibers does not change significantly across the length of the sample (no branches).

The second metric measures the similarity between two tractograms. Such a metric is useful to compare tractograms generated using different algorithms and/or parameters on the same dataset. For this task, we propose a distance metric based on closest neighbors, similar to other metrics described in the literature^41^^,^^42^ to compare individual streamlines. Briefly, to compare tractogram A with tractogram B, we consider each streamline in tractogram A and record the Euclidean distance to its closest neighboring streamline in tractogram B. Subsequently, the mean value across all streamlines in tractogram A is computed (referred to as ${\text{dist}}_{A\text{-}B}$). Since this metric is not symmetric (${\text{dist}}_{A\text{-}B}\neq {\text{dist}}_{B\text{-}A}$), we repeat the computation by comparing tractogram B to A and report the mean of the two values as our similarity metric. This method can be time-consuming if applied across thousands of streamlines (results in 200 million comparisons if the tractograms have 10,000 streamlines each). To mitigate this issue, we first simplify both tractograms by running a streamline clustering algorithm, Quickbundles,^41^ and only consider the cluster centroids. In addition, distances among the centroid streamlines are only considered if they arise from the same ROI in the mask image. We refer to this metric as the mean closest neighbor distance (${\mathrm{MCN}}_{\text{dist}}$).

### 2.4 Image Segmentation Pipeline

In addition to the tractography approaches described above, a complementary image segmentation tool is also developed and utilized in this work. A 2D U-net-based^43^ image segmentation network is implemented to perform multi-class segmentation on the 3D-MUSE image stacks. The network is trained to segment fascicle and epineurium regions in the image. The network encoder uses weights initialized by pre-training on the ImageNet^44^ database. Images are downsampled to a size of 1024×768 from their original resolution of 4000×3000  pixels to fit in GPU memory while using a batch size of 4. The network is trained for 100 epochs, using manual annotations of every 100th slice in the stack as training data. The trained network is then applied to all images in the stack. In some cases, manual corrections are made on the predicted masks to ensure that all pixels inside the outer epineurium boundary are labeled to one of two classes, either fascicle or epineurium. This step guarantees that the generated masks are anatomically appropriate and suitable for downstream analysis.

## 3 Software Features

**Fig. 1:**
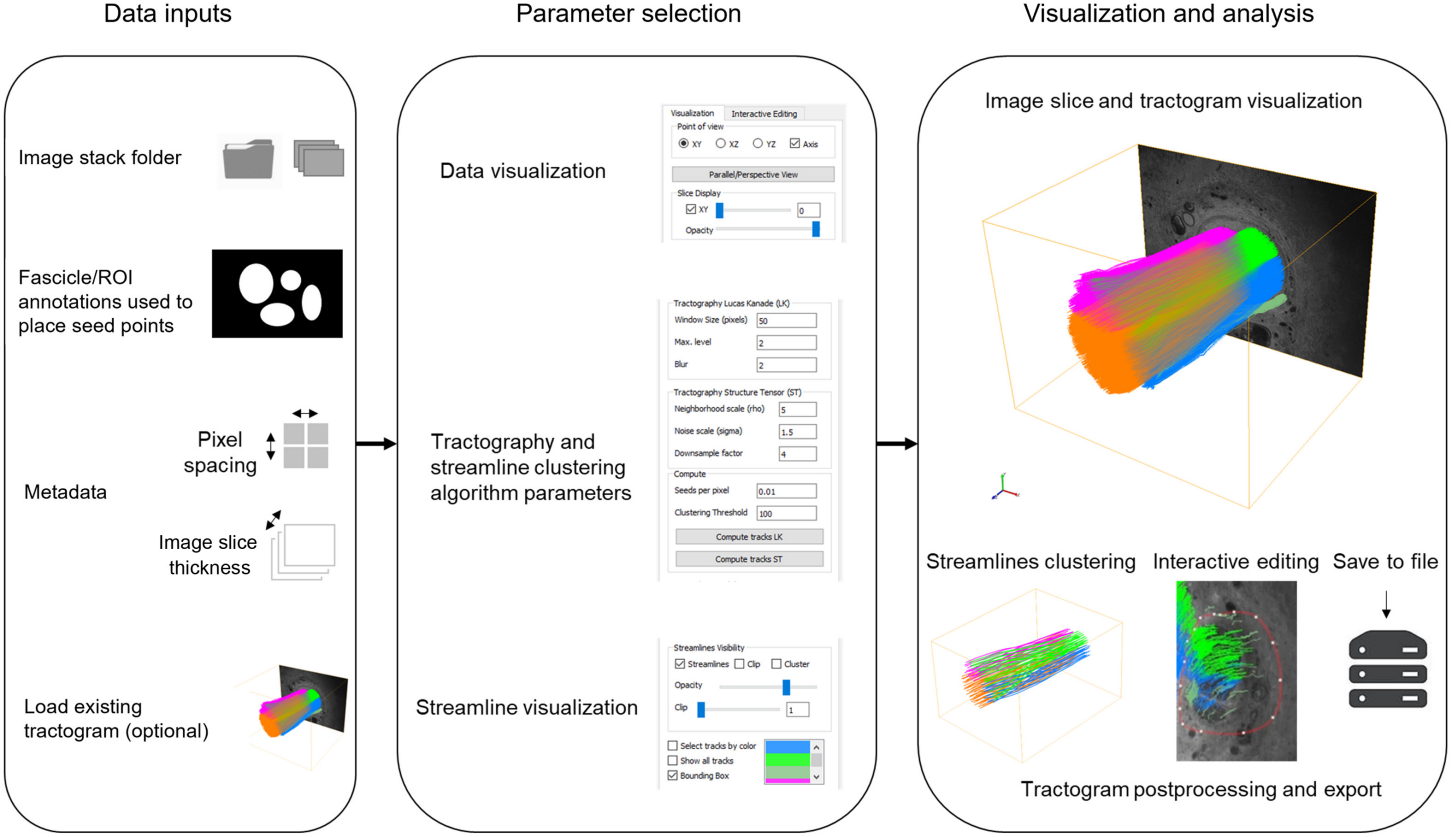
Workflow of the proposed software, NerveTracker, to visualize and track groups of nerve fibers in 3D-MUSE datasets. The software takes the image stack, manually delineated ROIs on the first slice, and image metadata as input; runs the chosen tractography algorithm; and provides interactive visualization and editing tools for the generated tractogram.

NerveTracker allows automated analysis of nerve tracts in 3D-MUSE images of peripheral nerves. The workflow outlining the software is presented in Fig. 1. In the upcoming text, we describe software features in greater detail, but some of the main features are summarized here:

• tractography using either optic flow or structure tensor methods
• create tracts from seeds within a user-supplied mask image
• visualize a specific image plane and tractogram in 3D
• interactive editing for manual corrections to the tractogram
• constrain the generated tractogram using fascicle segmentations
• cluster streamlines to generate representative tracts.

The software is written in Python and uses Qt v5.0 (Riverbank Computing, England, United Kingdom) for its GUI implementation and Visualization Toolkit 9.2 (VTK, Kitware Inc., Clifton Park, New York, United States) for 3D visualization. Upon initialization, appropriate file paths to the image stack and mask are provided. Imaging parameters such as pixel size, section thickness, and number of images in the stack are provided by means of a dialog window or a pre-defined XML file. The expected format and fields in the XML file are provided in Appendix A. The parameters of the tractography algorithm are set as desired and the flow estimation algorithm can be run in either direction of the stack. Using the UI, individual visualization elements (image slice, streamlines, cluster centroids, etc.) can be added to or removed from the visualization window as desired.

We implemented NerveTracker to provide simple image visualization for large (10 GB and greater) 3D-MUSE image volumes. The software currently supports 2D grayscale images in PNG format. We note that our method can routinely generate datasets that exceed system memory. For example, considering a 12-megapixel camera sensor and a single tile, 4000 image slices at 8-bit pixel depth would take up ∼50  GB of space. To reduce memory requirements on the user’s computer, the software does not read all images into memory. Instead, a virtual stack is created by reading only the requested 2D image slice from the disk at any time. This approach allows us to significantly reduce memory requirements during visualization. The tractogram and an arbitrary image slice can be viewed at the same time.

The software also considers memory constraints when running the tractography analyses. Since the optic flow method computes flow between two frames at a time, images can be loaded into memory directly. However, the structure tensor approach requires a 3D volume as input to estimate flow at each voxel. Since loading the full stack would not be possible in computers with limited memory, we process the stack one volume chunk at a time to ensure that the program does not request memory beyond available RAM. The number of slices in a processed chunk is provided as input along with other imaging metadata. Individual chunks are created with overlap, which is computed based on the user-defined neighborhood and noise scale parameters. This ensures consistency in vector calculations at pixels near the edge of a chunk.

The starting coordinates of the generated streamlines are initialized from the user-provided binary mask. A connected-components analysis is run to identify individual ROIs from the annotated image. Next, for each connected component, a list of all pixel coordinates inside the component is created. The list is randomly permutated, and seed points are selected starting from the first index in the list until the desired sampling density is reached. The default sampling density is set to 1 seed per 100 pixels and has a maximum value of 1 seed per pixel. We ensure that the seed point coordinates are the same between runs by setting the same seed value to the random generator before permuting the list. This allows an accurate comparison of tractograms generated with varying algorithm parameters.

Interactive editing of generated streamlines is achieved using the vtkContourWidget class, allowing the user to either create new streamlines or delete unwanted streamlines. Furthermore, the user can provide a corresponding stack of fascicle masks to constrain the tractography result. Since peripheral nerve fibers are always contained within fascicles, tracking of streamlines can be terminated if the tracts exit the provided fascicle mask. In addition, streamlines can be clustered using the Quickbundles algorithm, developed for diffusion MR datasets. Our implementation directly uses the DIPY Python package^36^ where this functionality is readily available.

## 4 Results

Tractography results are presented on two representative 3D-MUSE datasets. The datasets include a human cervical vagus nerve (sample 1) and a branch of the tibial nerve (sample 2). Both samples were sectioned at 3  μm thickness and imaged either with 0.9  μm pixel spacing (sample 1) or 0.74  μm pixel spacing (sample 2). Images are collected using a 12-megapixel camera, with image sizes of 4000×3000  pixels. Further details on sample preparation and imaging system specifications are provided in Appendix B. A total of 1500 images were collected for sample 1, and 900 images were collected for sample 2, giving ∼16 and 10 GB after conversion to grayscale, respectively. We found that the nerve in sample 2 covered approximately half of the image field of view; thus, an additional cropping step was applied to reduce the image size to around 2200×2400  pixels.

**Fig. 2:**
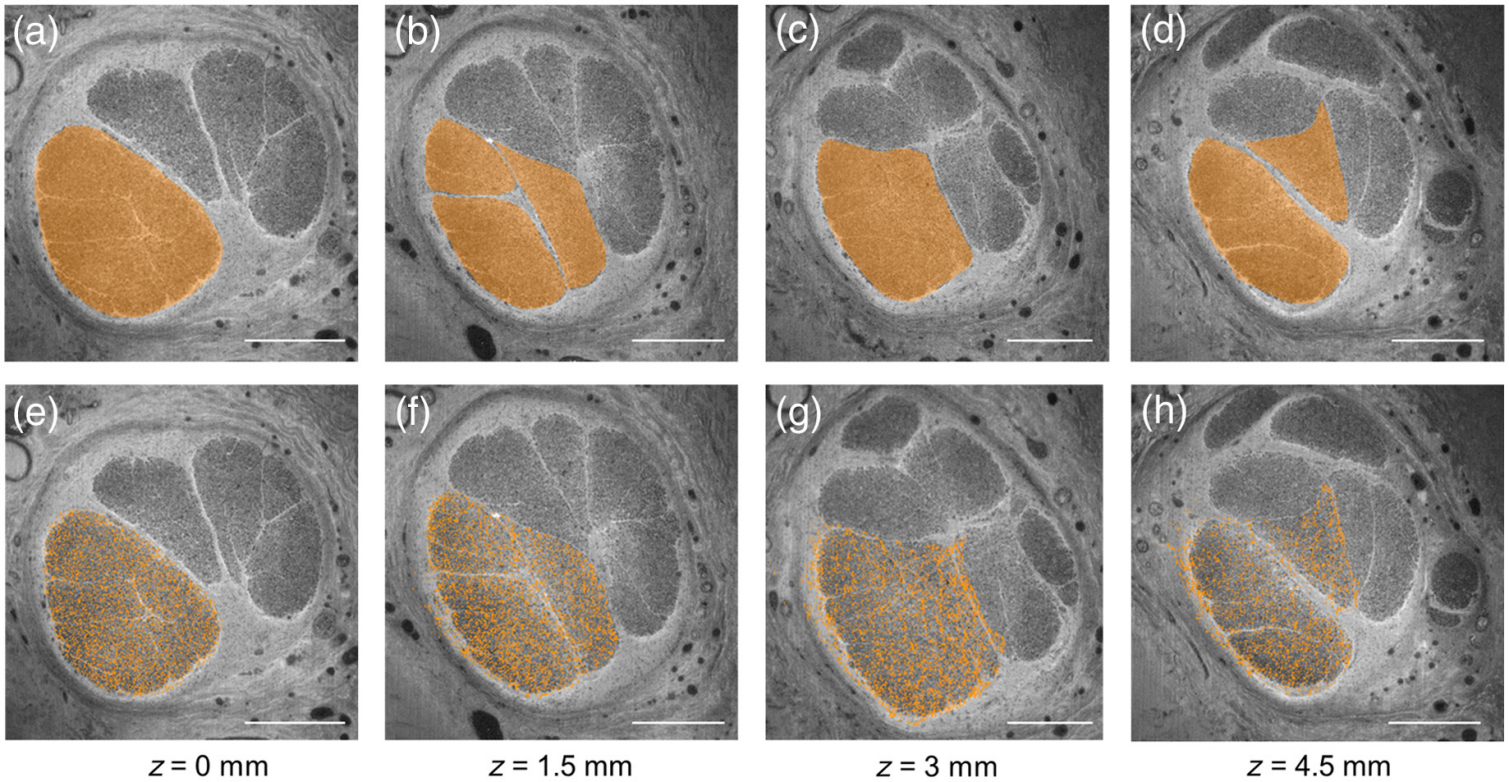
Correspondence between manual segmentation of fiber bundles and tractography results on select slices from sample 1. (a)–(d) Fibers within a select fascicle were outlined as a group and visualized with an orange mask as an overlay. These fibers were found to split into two separate fascicles at the last slice of the stack, shown in panel (d). (e)–(h) Tractography with optic flow analysis initiated at the first slice (z=0  mm) generates streamlines that resemble the manual segmentation result. The ${\text{Dice}}_{\text{norm}}$ metric for the orange streamlines was 0.88, 0.88, and 0.81 at z=1.5, 3, and 4.5 mm, respectively. Scalebars indicate 500  μm.

**Fig. 3:**
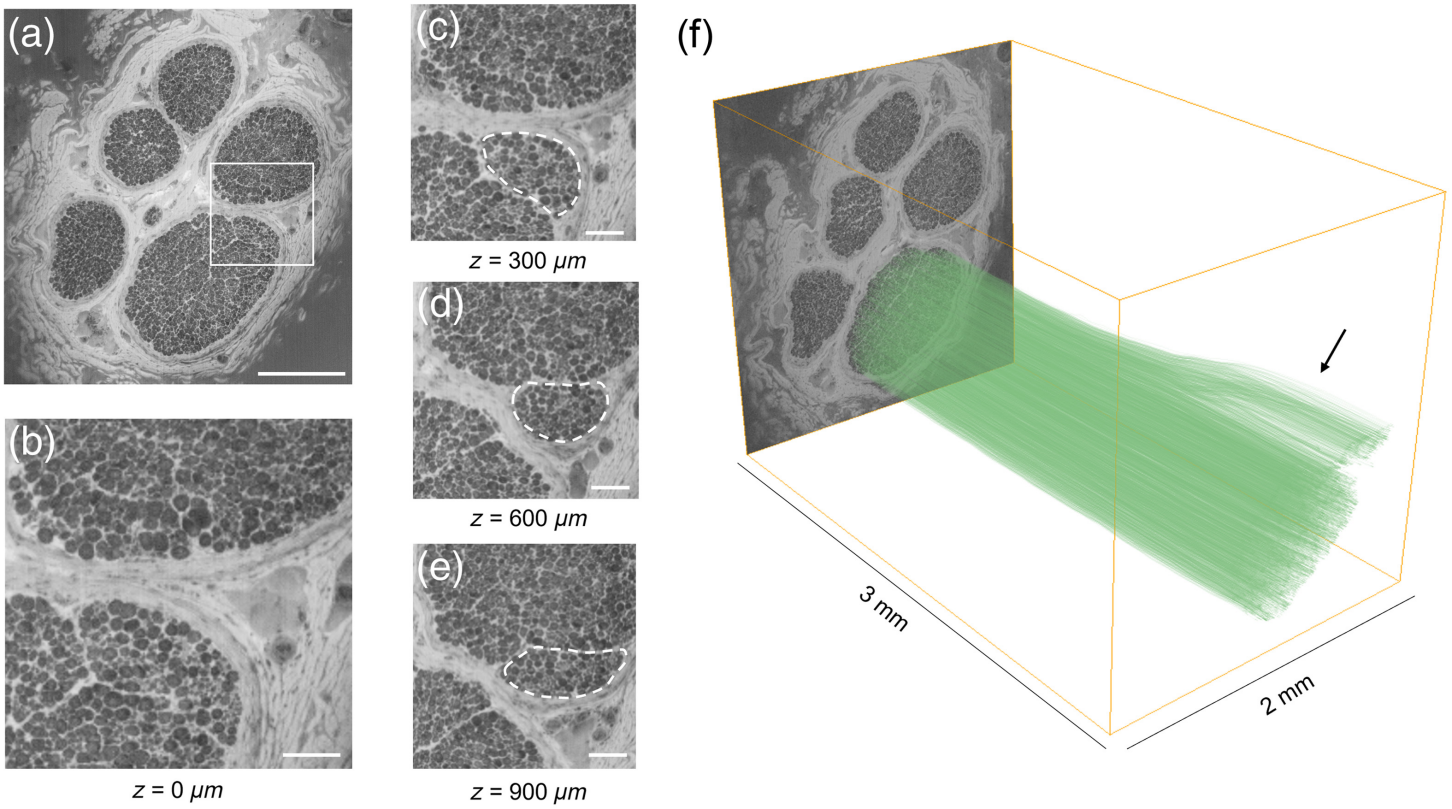
Tractography methods on 3D-MUSE data can detect the movement of fiber groups from one fascicle to another. (a) 3D-MUSE image slice (green channel, contrast adjusted) from sample 2, illustrating several large fascicles. We consider this slice to be at z=0  μm for the purpose of this illustration. (b) Close-up view of the ROI outlined by the white box in panel (a). (c)–(e) Images at different z depths in the image stack, all cropped at the same ROI. A group of fibers can be identified moving from the fascicle below to the fascicle on top, indicated by a dashed outline. (f) 3D visualization of the tractogram generated by structure tensor analysis shows a split in the fiber groups (black arrow) corresponding to this moving group of fibers. Scalebars correspond to 500  μm in panel (a) and 100  μm in panels (b)–(e). (Video 2, MP4, 24.1 MB [URL: https://doi.org/10.1117/1.JBO.29.7.076501.s2])

To verify that the tractography output matches the spatial organization of fiber groups in the image stack, we created ground truth segmentations on select slices and computed the ${\text{Dice}}_{\text{norm}}$ metric. For sample 1, we manually traced the outline of fibers in a particular fascicle on the first image slice. A flythrough video of the stack along with the segmentations overlaid is shown in Video 1, MP4, 53.0 MB [URL: https://doi.org/10.1117/1.JBO.29.7.076501.s1]. We consider four slices at equally spaced intervals along the stack and overlay the manual tracing as an orange mask [Figs. 2(a)–2(d)]. From these segmentations, we found that this group of fibers merged with other fascicles and eventually split across two fascicles when they reached the last image of the stack [Fig. 2(d)]. In Figs. 2(e)–2(h), the result of the tractography analysis using the optic flow method is overlaid on the raw images for comparison. The tractography analysis is found to match the ground truth segmentation to a large extent when tracking both in the forward (Fig. 2) and backward (Fig. S1 in the Supplementary Material) directions. A similar analysis was performed for sample 2 as well. The mean ${\text{Dice}}_{\text{norm}}$ values across all bundles for both samples are provided in Table 1. We find that this metric is always above 0.66 and has a mean value over 0.75. In addition, we find that the structure tensor approach seems to perform better on both datasets compared with the optic flow analysis. Along with the quantitative analysis, we qualitatively verified that the tractogram is consistent with respect to the imaging data for sample 2. In this sample, a group of fibers are outlined as they exit one fascicle and enter another [Figs. 3(a)–3(e)]. In Fig. 3(f), we selectively visualize streamlines initiated from the fascicle of interest and identify the branching event, with a group of streamlines exiting from the bundle, corresponding to the image data. Video 2 demonstrates interactive visualization of this event in the software by moving across the image stack and viewing from various camera angles.

**Table 1:** ${\text{Dice}}_{\text{norm}}$ values measuring overlap between ground truth segmentation of fiber bundles and tractograms at intermediate slices in the stacks (1/3_rd_, 2/3_rd_, and at the end of the stack).

**Sample 1**	***z*** = **1.5 mm**	***z*** = **3 mm**	***z*** = **4.5 mm**	**Mean across slices**
Optic flow	0.87	0.74	0.66	0.76
Structure tensor	0.90	0.79	0.67	0.79
**Sample 2**	***z*** = **0.9 mm**	***z*** = **1.8 mm**	***z*** = **2.7 mm**	**Mean across slices**
Optic flow	0.94	0.90	0.88	0.91
Structure tensor	0.96	0.93	0.91	0.93

The metric is computed from the Dice score on a specific slice and normalized with respect to the Dice score of the image slice containing the seed points. In these experiments, seed points were placed on the first slice of the image stack; thus, ${\text{Dice}}_{\text{norm}}$ is equal to 1 at z=0  mm. The normalization step is performed to account for variations in seed point density, where a higher density of seed points can result in a greater Dice coefficient.

**Fig. 4:**
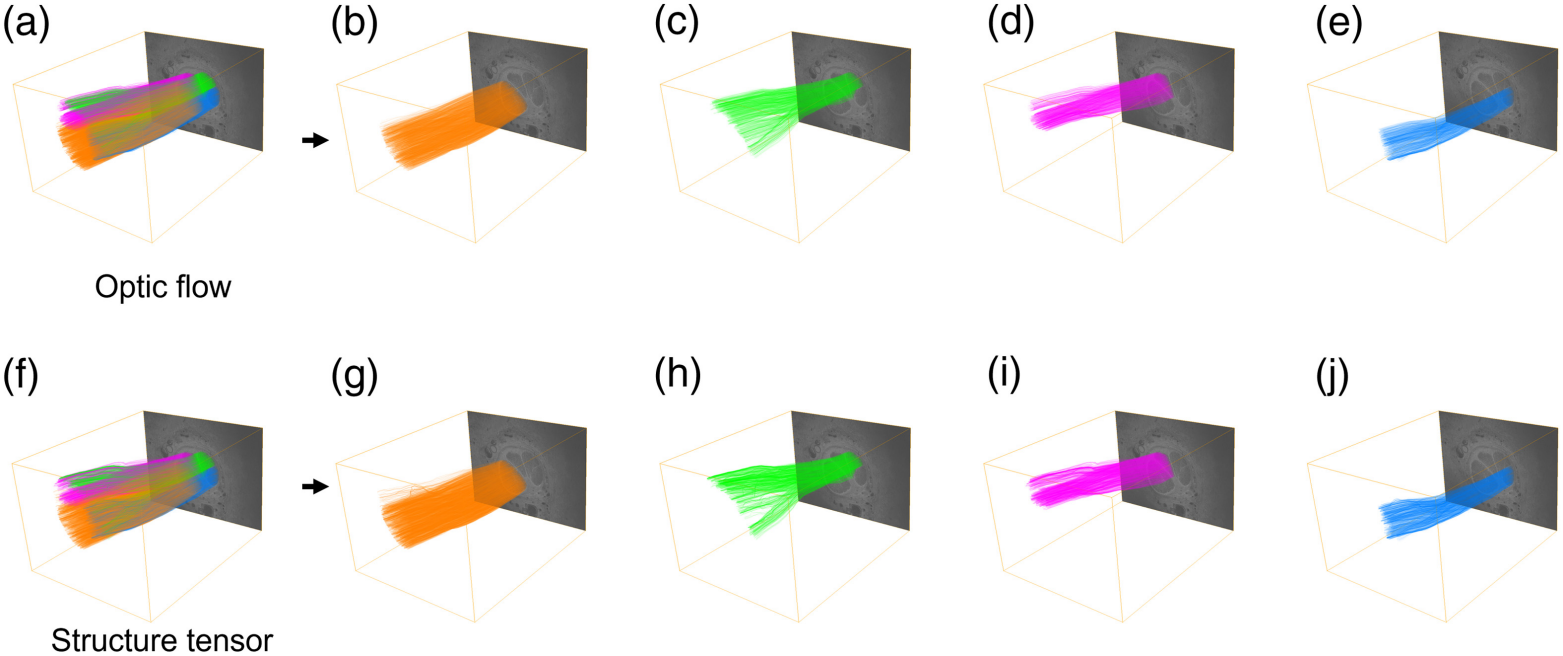
Comparison of tractogram results generated from optic flow (a)–(e) and structure tensor (f)–(j) analysis. To aid the visualization of dense tractograms (a), (f), tracts originating from individual fascicles or ROIs are rendered with unique colors. This allows selective visualization of individual bundles, as shown in panels (b)–(e) and (g)–(j) for the optic flow and structure tensor approaches, respectively. We find that both methods provide visually similar results on both datasets.

To further identify differences in the output of the two tractography approaches, we performed both qualitative and quantitative comparisons of the generated streamlines. In Fig. 4, we visualize streamlines generated both from optic flow and structure tensor analysis on sample 1. Comparing all streamlines in Figs. 4(a) and 4(f), we did not identify any significant differences. However, since the generated tractograms are dense due to a large number of streamlines (nearly 15,000), we show streamlines originating from individual fascicle bundles arranged by decreasing fascicle areas in Figs. 4(b)–4(e) and 4(g)–4(j). The overall structure of the streamlines was found to be similar across all fascicles. We computed the ${\mathrm{MCN}}_{\text{dist}}$ metric between the two tractograms and found it to be around 49  μm, indicating good similarity since it is less than 2% of any dimension of the image stack. The results were comparable when a similar analysis was done for sample 2, as shown in Fig. S2 in the Supplementary Material. The ${\mathrm{MCN}}_{\text{dist}}$ metric between the optic flow and structure-tensor-based tractograms for this sample was found to be around 100  μm (less than 6% of any dimension of the stack). Parameters used in the optic flow and structure tensor analyses are provided in Appendix C. In terms of computation time on 1500 slices at a 4000×3000 image size, generating the tractograms took around 14 min for the optic flow analysis and 18 min for the structure tensor approach.

We tested whether downsampling our datasets would have a significant effect on the generated tractograms. We took the image stacks acquired at 3  μm image slice thickness and dropped one or three slices in between to effectively create stacks at 6 and 12  μm image slice thickness. Alternatively, images were downsampled in-plane (XY) by a factor of 2 or 3. We computed the mean ${\text{Dice}}_{\text{norm}}$ metric as described previously and show the mean value across intermediate slices in Table 2. We found that the overlap metrics for the downsampled stacks and the baseline stack are similar (∼2% to 9% change). We also computed the ${\mathrm{MCN}}_{\text{dist}}$ between the tractograms from the downsampled and baseline stacks and found that these values also indicate high similarity (Table 3).

**Table 2:** Quantifying changes in tractogram accuracy with respect to changes to image properties.

**Sample 1**	**Baseline**	**2**× **downsampling in** ***Z***	**4**× **downsampling in** ***Z***	**2**× **downsampling in** ***XY***	**3**× **downsampling in** ***XY***
Optic flow	0.76	0.77	0.75	0.79	0.77
Structure tensor	0.79	0.80	0.79	0.81	0.78
**Sample 2**	**Baseline**	**2**× **downsampling in** ***Z***	**4**× **downsampling in** ***Z***	**2**× **downsampling in** ***XY***	**3**× **downsampling in** ***XY***
Optic flow	0.91	0.93	0.94	0.90	0.83
Structure tensor	0.93	0.93	0.93	0.94	0.93

We modify image characteristics to see the effect on corresponding tractograms. We consider the original stack at 3  μm image slice thickness as the baseline tractogram. We test stacks with larger z spacing by removing alternate slices from the stack to generate stacks with 6 and 12  μm image slice thickness (2× and 4× downsampling). In addition, we tested the effect of downsampling the images in XY (2× and 3×). As in Table 1, ${\mathrm{Dice}}_{\text{norm}}$ values were computed at three locations in the stack (1/3_rd_, 2/3_rd_, and at the end of the stack). The mean value is reported here.

**Table 3:** ${\mathrm{MCN}}_{\text{dist}}$ values (in μm) provide a measure of similarity between two tractograms.

**Sample 1**	**2**× **downsampling ** **in** ***Z***	**4**× **downsampling ** **in** ***Z***	**2**× **downsampling ** **in** ***XY***	**3**× **downsampling ** **in** ***XY***
Optic flow	29	37	41	50
Structure tensor	45	64	43	37
**Sample 2**	**2**× **downsampling ** **in** ***Z***	**4**× **downsampling ** **in** ***Z***	**2**× **downsampling ** **in** ***XY***	**3**× **downsampling ** **in** ***XY***
Optic flow	18	25	39	44
Structure tensor	25	33	40	37

As in Table 2, the original image stack at 3  μm image slice thickness is the baseline stack. The numbers indicate ${\mathrm{MCN}}_{\text{dist}}$ values when compared with the baseline tractogram.

Post-processing operations on generated streamlines include streamline clustering to create representative streamlines. Although the software allows the user to specify a seed point sampling density as well as an opacity value for the rendered streamlines, these settings may not be ideal for visualization. For example, in Figs. S3(a)–S3(c) in the Supplementary Material, reducing the seed point sampling density yields a less dense tractogram but could miss tracking some regions within fascicles in the image. Similarly, images in Figs. S3(d)–S3(f) in the Supplementary Material show the effect of varying opacity. A visualization with 10% streamline opacity does not provide significant improvements compared with higher values, and further reductions would reduce the overall visibility of the streamlines. Instead, by applying a clustering algorithm on the streamlines (Quickbundles), the user can get a compact representation of the tractogram both in terms of visualization as well as file size. The result of applying the clustering algorithm to a tractogram is shown in Fig. S4 in the Supplementary Material. The clustering algorithm is also used during the computation of the ${\mathrm{MCN}}_{\text{dist}}$ metric to compare two tractograms.

**Fig. 5:**
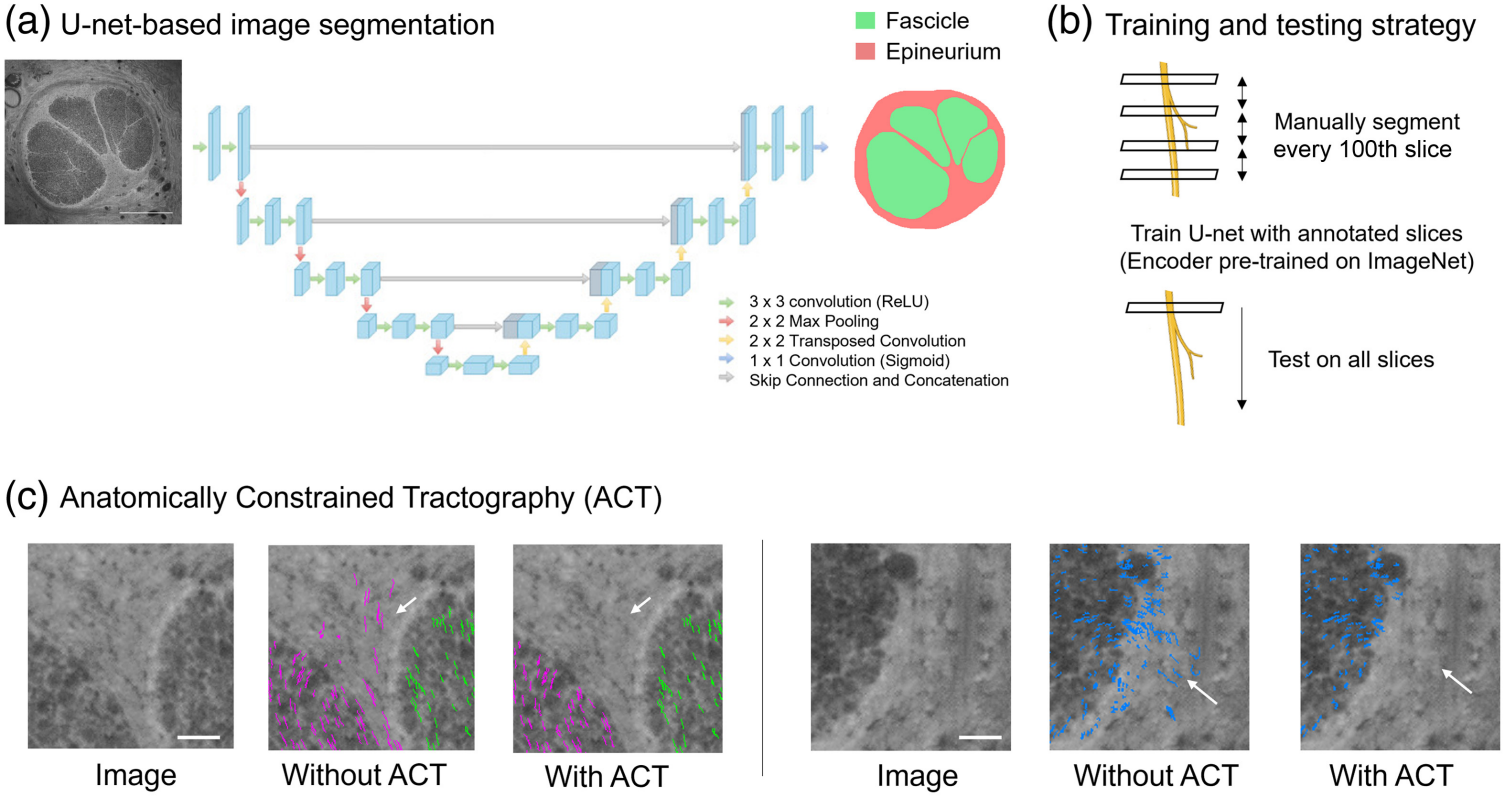
Deep-learning-based image segmentation pipeline for segmenting fascicles and epineurium regions in 3D-MUSE images. Since the method can generate many 2D slices, manual annotation of each slice is time-consuming. Thus, we segment a few slices in the stack (1 out of 100) and train a model to subsequently segment all slices in the stack. (a) U-net architecture used in this work, (b) training and testing strategy employed in this work, and (c) modifications to tractography result when using the segmentation masks. Video 5 shows a flythrough video of the segmentation result for this dataset. Images in panel (b) were created with BioRender. Scale bars in panels (a) and (c) indicate 500 and 30  μm, respectively. (Video 5, MP4, 21.5 MB [URL: https://doi.org/10.1117/1.JBO.29.7.076501.s5])

In addition, the software provides a few streamline editing tools to the user. Streamlines can be edited by drawing a contour over a specific image slice and choosing to either create new streamlines or delete existing streamlines as shown in Video 3, MP4, 68.5 MB [URL: https://doi.org/10.1117/1.JBO.29.7.076501.s3] and Video 4, MP4, 27.3 MB [URL: https://doi.org/10.1117/1.JBO.29.7.076501.s4], respectively. In terms of automated editing, the user can provide fascicle masks for each image in the stack as described in Figs. 5(a) and 5(b). The software can use these masks to remove streamlines that exit the fascicle regions. This process is referred to as anatomically constrained tractography (ACT),^45^ and the results on two image slices from sample 1 are shown in Fig. 5(c). We implement a method for automatic fascicle segmentation with a 2D U-net network separately, outside the current software package. The results of running the segmentation algorithm on the image stack from sample 1 are shown in Video 5.

## 5 Discussion and Conclusion

To our knowledge, this is the first study demonstrating microscopic tractography on human peripheral nerves. This has been made possible by our new software (NerveTracker), which tracks groups of nerve fibers in 3D-MUSE images. Below, we review the algorithms and results and describe some future directions.

We proposed a metric based on the Dice coefficient to measure the accuracy of the tractograms compared with manual ground truth segmentations of fiber groups. We obtain point clouds at each 2D slice in the tractogram and convert them into a binary mask to compute a Dice overlap metric with the ground truth segmentations. We found that the ${\text{Dice}}_{\text{norm}}$ metric was greater than 0.75 on average, for both the optic flow and structure tensor approaches on both datasets. Since a Dice score of 1 indicates perfect correspondence, the values obtained here show that the tractograms are reasonably accurate. Sample 2 was found to have higher ${\text{Dice}}_{\text{norm}}$, possibly due to better contrast from the large diameter fibers in the tibial nerve. We also identified that the tractograms were robust to changes in pixel size and section thickness when evaluated with the ${\text{Dice}}_{\text{norm}}$ metric. We identified that the tractograms retain their accuracy at both 6 and 12  μm image slice thickness. This indicates that instead of capturing an image of the block face after every 3  μm slice, the system could skip imaging a few slices and still provide suitable tractograms. This approach could substantially reduce overall imaging time. A similar result was also found with downsampling in plane by a factor of 2 and 3, suggesting that a reduction in system magnification (leading to requiring fewer image tiles) may be possible. Alternate distance-based metrics acting on point clouds (e.g., chamfer distance^46^) could also be considered in future studies.

We found that both methods for estimating fiber orientation generated visually similar tractography results. However, from the ${\text{Dice}}_{\text{norm}}$ metric, structure tensor gave somewhat better results than optic flow. This is reasonable since the structure tensor approach takes several image slices into consideration when estimating flow vectors, as compared with the optic flow approach, which tracks points from one image frame to the next. In addition, since the optic flow algorithm was run with a window size of 50 pixels at two resolution levels (i.e., the original resolution and 2× downsampling), the largest field seen by the algorithm was 100×100  pixels at the original resolution. Considering an average fiber diameter of ∼5  μm, this field may hold up to 20 fibers. However, fiber groups that branch out from a fascicle may be smaller than the window; thus, structures outside the branching fiber bundle and still inside the window could influence the flow calculations.

Deep learning-based image segmentation methods were successfully applied to segment fascicles and epineurium in the cross-sectional nerve images. Since a single 3D-MUSE stack can contain over 1000 2D images, manual annotation of the slices is time consuming and not readily feasible. We adopted a strategy of selectively labeling every 100th slice within a stack, trained a network on those slices, and predicted segmentations for all slices in the stack. This approach is suitable since we observe significant variation in staining intensity and contrast across samples due to the nature of whole-mount staining. We are continuing to test other suitable network training strategies as well as newer model architectures.^47^ We created methods in NerveTracker to take such segmentations as input and restrict streamlines to stay within the fascicle regions, thereby ensuring biologically feasible tracts. The segmentations can also be used to generate accurate anatomical mesh models for computational modeling studies of neuromodulation.^48^

Data from complementary imaging methods can be incorporated with our microscopic tractograms. For example, information on fiber type (afferent versus efferent) could be accessed by suitable immunohistochemistry methods on thin sections.^49^ By combining such complementary information of fiber types on the tractogram, it would be possible to create a more functionally relevant map of the nerve. Similarly, by incorporating EM, it should be possible to identify the distribution of both myelinated and unmyelinated fibers within a peripheral nerve cross-section. Analyses can also be extended to imaging modalities having a lower resolution, but larger field of view. For example, microscopic tractography could help us further understand fascicular organization as seen in micro-CT.^11^ Similarly, our approach could be complementary to lower-resolution diffusion MRI tractography. It could provide validation for higher-resolution diffusion MRI as they continue to develop the technology.

Some extensions to our methods are possible. Further tests are required to determine if our approach can be extended to higher-resolution micro-CT or light-sheet datasets. We are encouraged in this regard as others have reported promising results using the structure tensor approach on such data.^38^^,^^50^ Alternatively, deep learning-based methods such as FlowNet and MMFlow have been proposed for the task of object tracking in video sequences. It should be possible to implement and test these methods in a straightforward manner with NerveTracker. We have currently implemented lightweight visualization of graphical tractograms and individual image slices. Given the large data sizes (∼100  GB), routine 3D rendering of 3D-MUSE image data would be difficult. A promising method for handling large data is formats that store data at multiple resolutions (e.g., Zarr). We could include volumetric visualizations of such datasets when the core VTK library supports them in future software releases. When the VTK library supports volume rendering options for such file formats, we could include them in future software releases.

In summary, this work presents a software toolkit for visualizing and tracking fiber groups in block-face microscopy datasets. We found that 3D-MUSE allows volumetric imaging of peripheral nerves, and the software presented here can generate useful insights from this relatively new imaging approach. The software is written in Python and is thus easily customizable if new features are to be added. The software currently supports fast visualization of 2D image slices and implements two flow estimation algorithms and several postprocessing and analysis methods on the generated tractograms. The computation time for the tractography analyses was found to be reasonable (around 15 min for generating streamlines on a dataset of 1500 images), suggesting the suitability of the methods to process larger datasets. Interactive streamline editing tools include both automated and manual correction of streamlines. The software presented here can be used as an alternative to track objects or features of interest in 3D image stacks, without the need for explicitly segmenting them on each slice.

## 6 Appendix A

The image metadata file should contain the following fields:

a. pixel_size_xy: pixel size in physical dimensions (microns).
b. image_slice_thickness: distance between consecutive images in the stack (microns).
c. image_type: file type of the images (currently accepts .png or .zarr)
d. num_images_to_read: number of images to load into the software, should be less than or equal to the number of images present in the chosen image folder
e. step_size: number of images in each chunk of data provided to the structure tensor analysis algorithm

An example XML metadata file is provided below.

<?xml version="1.0"?>

<root>

 <pixel_size_xy name="0.9"/>

 <image_slice_thickness name="3"/>

 <image_type name=".png"/>

 <num_images_to_read name="1000"/>

 <step_size name="64" />

</root>

## 7 Appendix B

Nerve tissues were prepared for 3D-MUSE imaging using a whole-mount staining and embedding approach, similar to the protocol described in our previous work.^24^ First, samples were stained with Ehrlich’s hematoxylin (12.5% v/v in DI water) to create absorptive contrast to nerve fibers and nuclei. To allow the stain to completely diffuse into the tissue, samples were left in the staining solution on a rocker for 4 days. Next, they were dehydrated through the increasing concentrations of ethanol (30%, 50%, 70%, 80%, 90%, and 100%, 4 h each). Rhodamine B was included in the last ethanol step (0.25% w/v) to create a contrast with hematoxylin. Subsequently, samples were placed in Glycol methacrylate (GMA) monomer for 1 day and followed by GMA infiltration solution for 2 days. Finally, they were placed in an embedding mold and polymerized by adding an initiator to the infiltration solution. The hardened blocks were trimmed and mounted on plastic chucks, making them suitable for sectioning on the microtome. The imaging system consisted of an infinity-corrected microscope with a Nikon objective (either 4×/0.13 NA or 5×/0.14 NA), tube lens (Thorlabs, Newton, New Jersey, United States, TTL-100-A), and a CMOS camera [Teledyne FLIR (Wilsonville, Oregon)]. The light source consisted of a UV light source (Thorlabs, M280L4) mounted on the imaging system to provide oblique illumination.

## 8 Appendix C

Unless otherwise specified, parameters used in the optic flow analysis are as follows. The window size was set to 50 pixels, and the number of resolution levels was set to 2. Prior to running the optic flow analysis, a Gaussian blur with a standard deviation (σ) of 2 pixels was applied to the images to reduce noise in the gradient computation. Similarly, the parameters used in the structure tensor analysis were a neighborhood scale of 5 pixels and a noise scale of 1 pixel. Images were down-sampled in XY by the closest integer factor to create near-isotropic voxels (2.7×2.7×3  μm for sample 1 and 2.96×2.96×3  μm for sample 2) prior to computing the structure tensor.

## Supplementary Material













## Data Availability

The software has been made available at https://github.com/ckolluru/NerveTracker. Example 3D-MUSE datasets will be made available upon reasonable request to the corresponding author.
