# Supplementary material for: NerveTracker: a Python-based software toolkit for visualizing and tracking groups of nerve fibers in serial block-face microscopy with ultraviolet surface excitation images
Source: J Biomed Opt. 2024 Jun 18;29(7):076501. doi: 10.1117/1.JBO.29.7.076501 (PMC11188586; doi:10.1117/1.JBO.29.7.076501)
Supplement: Supplementary file 1 [file JBO_029_076501_SD001.pdf]

Supplementary figures

NerveTracker: a Python-based software toolkit for visualizing and tracking groups of nerve fibers in serial block-face MUSE images

Chaitanya Kolluru[1], Naomi Joseph[1], James Seckler[1], Farzad Fereidouni[2], Richard Levenson[2], Andrew Shoffstall[1,3], Michael Jenkins[1,3,4], David Wilson[1,5]

[1]Department of Biomedical Engineering, Case Western Reserve University, Cleveland, OH 44106
[2]Department of Pathology and Laboratory Medicine, UC Davis Medical Center, Sacramento, CA 95817
[3]Louis Stokes Cleveland VA Medical Center, Cleveland, OH 44106
[4]Department of Pediatrics, Case Western Reserve University, Cleveland, OH, 44106, USA
[5]Department of Radiology, Case Western Reserve University, Cleveland, OH, 44106, USA

Corresponding author: david.wilson@case.edu

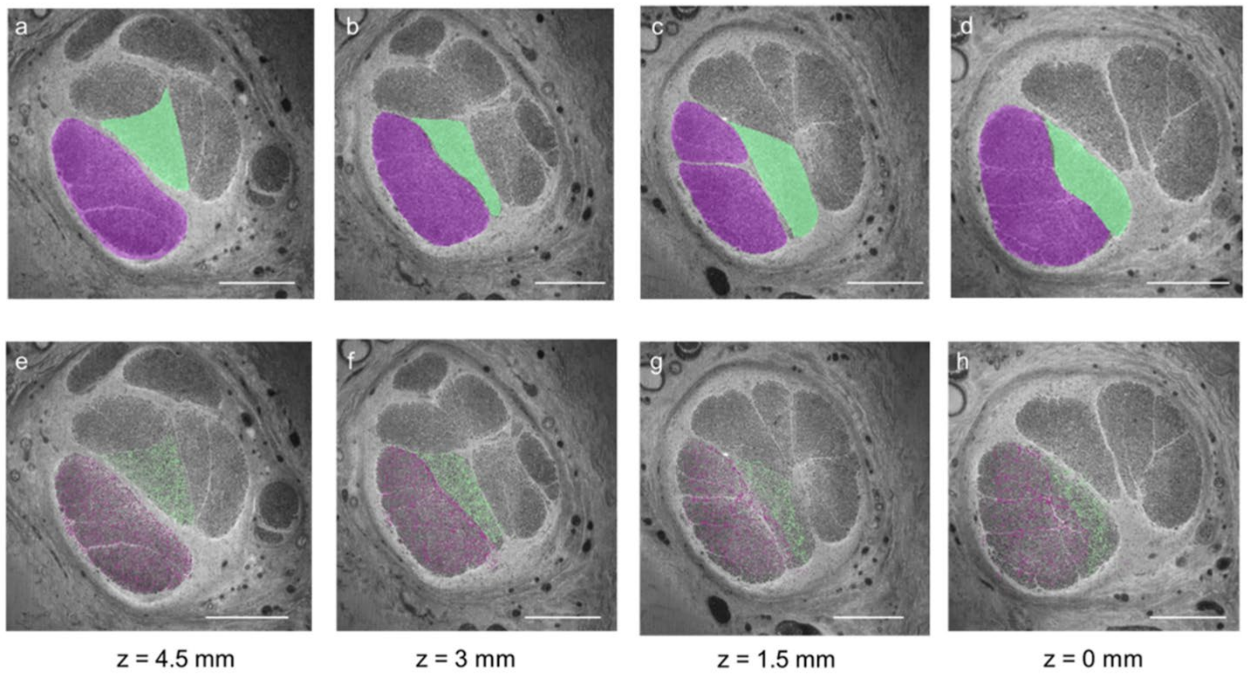

Supplementary Figure S1. Correspondence between manual segmentation of fiber groups and tractography output on select slices from sample 1, with tracking performed in the reverse direction compared to Figure 2. (a – d) Images and segmentation mask overlay, in the reverse order compared to Figure 2a– 2d. When tracking in reverse, fibers from two distinct fascicle bundles merge into one fascicle. (e-h) Tractography with optic flow analysis resembles the manual segmentation result, when compared with the spatial locations of the streamlines colored in purple and light green. Scalebars indicate 500 μm.

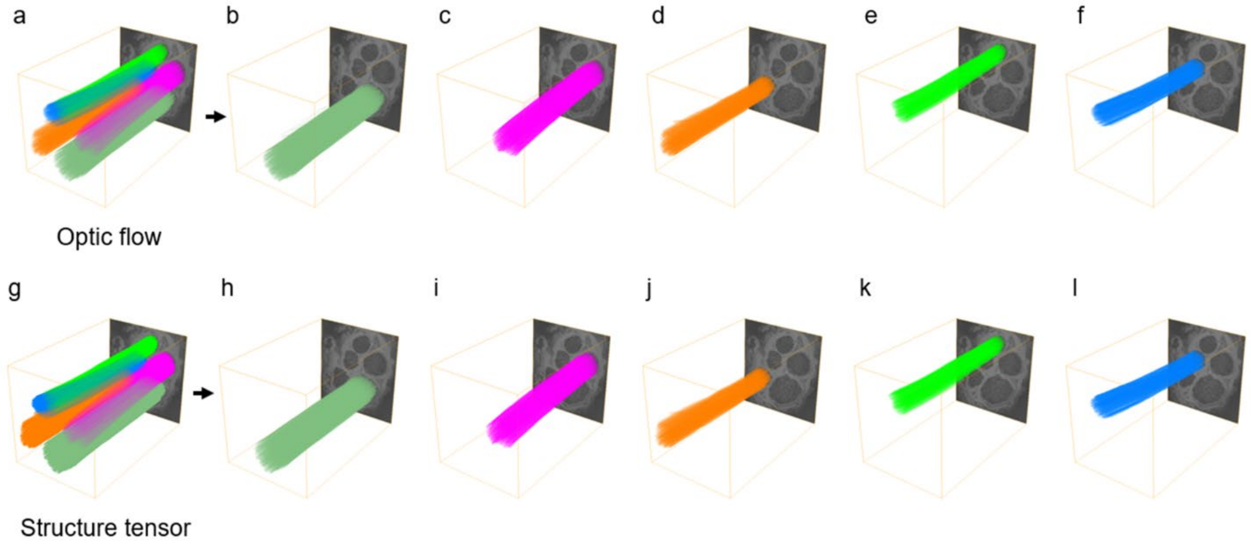

Supplementary Figure S2. Comparison of tractogram results generated from (a-f) optic flow and (g-l) structure tensor analysis for sample 2. Parameters used in both tractography analyses were the same as in Figure 4. We note that the results of the two flow estimation methods are visually similar for this sample as well.

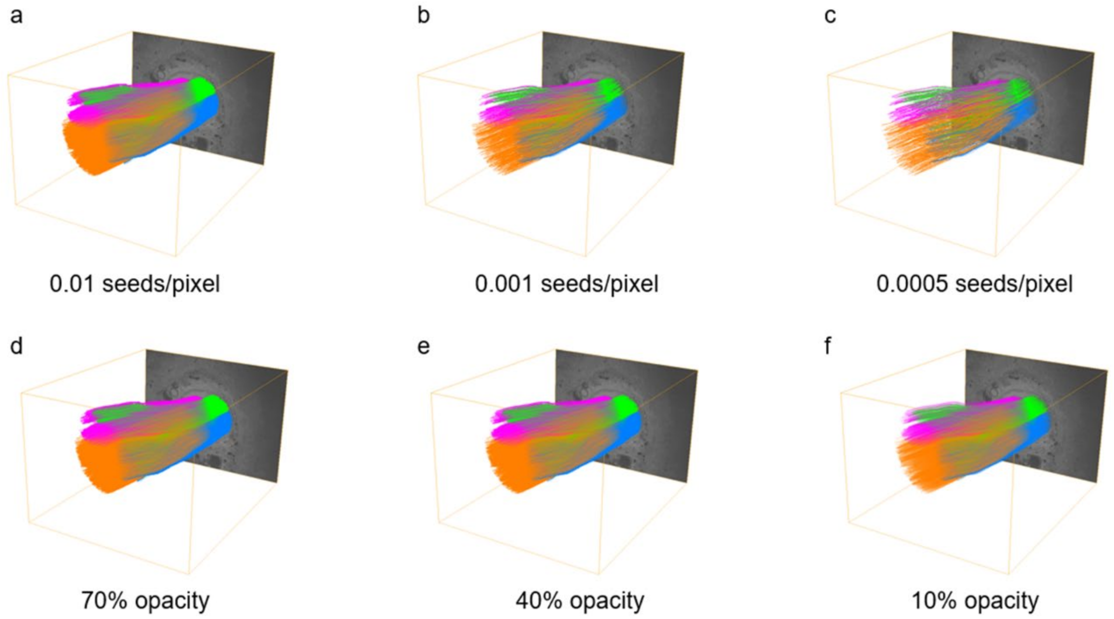

Supplementary Figure S3. Optic flow derived tractogram for sample 1, with modifications either in seed point sampling density or streamline opacity. (a – c) Tractograms created with varying seed point density on the first image slice: 0.01 seeds per pixel (a), 0.001 seeds per pixel (b), and 0.0005 seeds per pixel (c). (d – f) Opacity of streamlines can also be modified, tractograms with the following opacity values are shown for a chosen seed point sampling density (0.01 seeds/pixel): 70% (d), 40% (e), 10% (f).

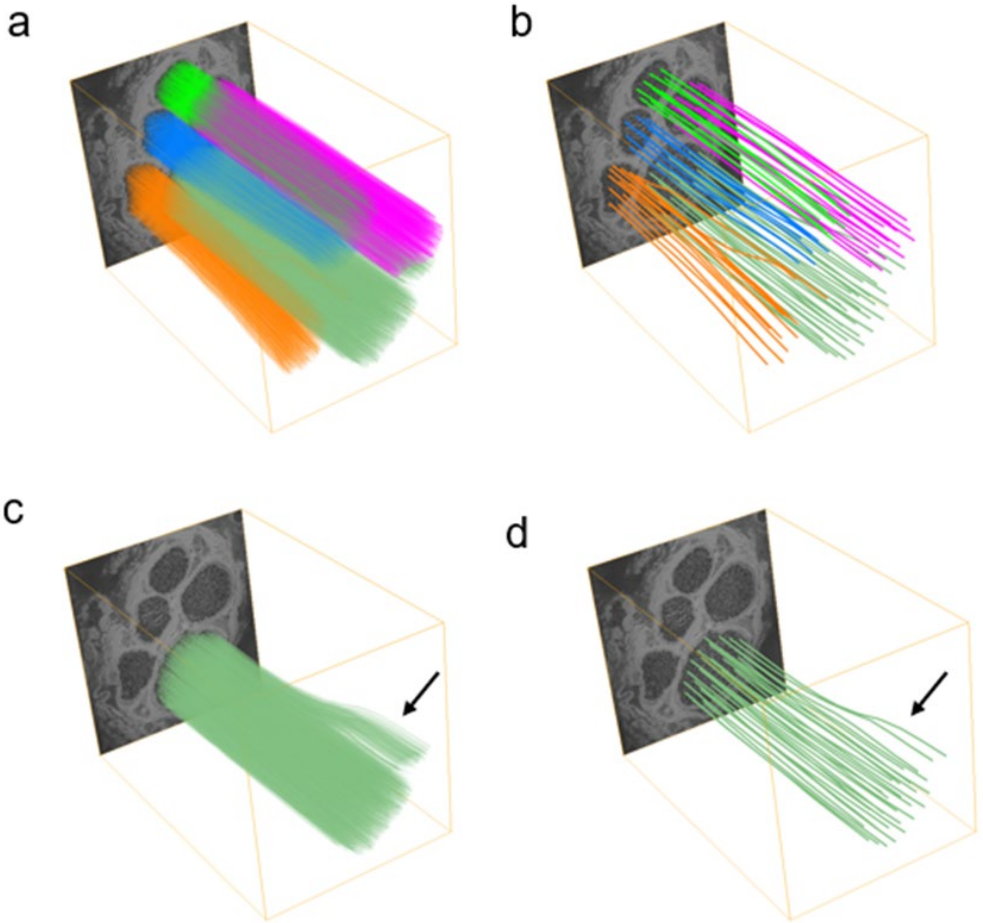

Supplementary Figure S4. Result of applying a streamline clustering algorithm, Quickbundles on a dense tractogram. (a) Tractogram generated by the optic flow approach with a seed point density of 0.01 seeds per pixel. (b) Cluster centroids generated by the clustering algorithm on tractogram from (a), created with a clustering threshold of 100 μm. (c) Visualization of a group of fibers originating from a specific ROI from the seed mask image (d) Cluster centroids of streamlines in (c) reveals that clustering at this distance threshold preserves the small group of fibers that exist this fascicle (illustrated by black arrows and in Figure 3).
